# A Disposable Nitinol Memory Alloy Anal Fistula Clip (AFC) for the Treatment of Cryptoglandular Fistula-In-Ano: a Prospective, Randomized, Controlled Study With Short-Term Follow-Up

**DOI:** 10.1007/s11605-022-05355-4

**Published:** 2022-05-25

**Authors:** Yaxian Wang, Yanlan Wu, Yehuang Wang, Bin Jiang, Chungen Zhou, Yang Zhang

**Affiliations:** 1 Nanjing Pukou Hospital of Traditional Chinese Medicine, Nanjing, 211800 Jiangsu Province China; 2 National Colorectal Disease Center of Nanjing Hospital of Chinese Medicine Affiliated to Nanjing University of Chinese Medicine, Nanjing, 210022 Jiangsu Province China

**Keywords:** Nitinol memory alloy anal fistula clip, Endorectal advancement flap, Cryptoglandular fistula-in-ano

## Introduction

**Fig. 1:**
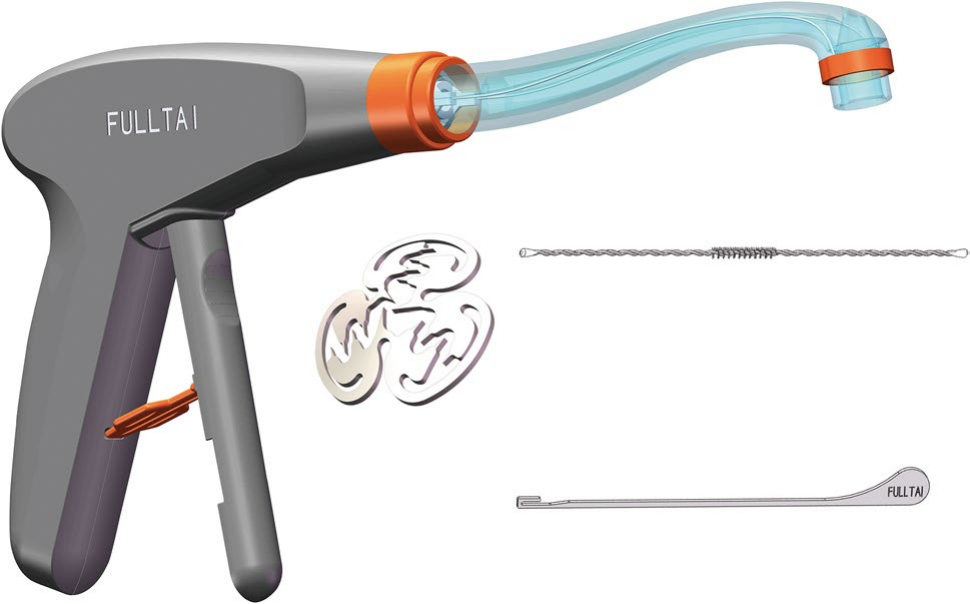
The clip is 14 mm in diameter and is made of a super-elastic shape memory alloy (nitinol). The clip system consists of the applicator and a special fistula brush

Surgical treatment of fistula-in-ano is an ongoing challenge and has led to the design of the disposable shape memory nitinol anal fistula clip (AFC) (Fig. 1). We conducted a prospective clinical study to evaluate the efficacy and safety of the AFC for cryptoglandular fistula-in-ano treatment compared with the endorectal advancement flap (ERAF).

## Methods

**Fig. 2:**
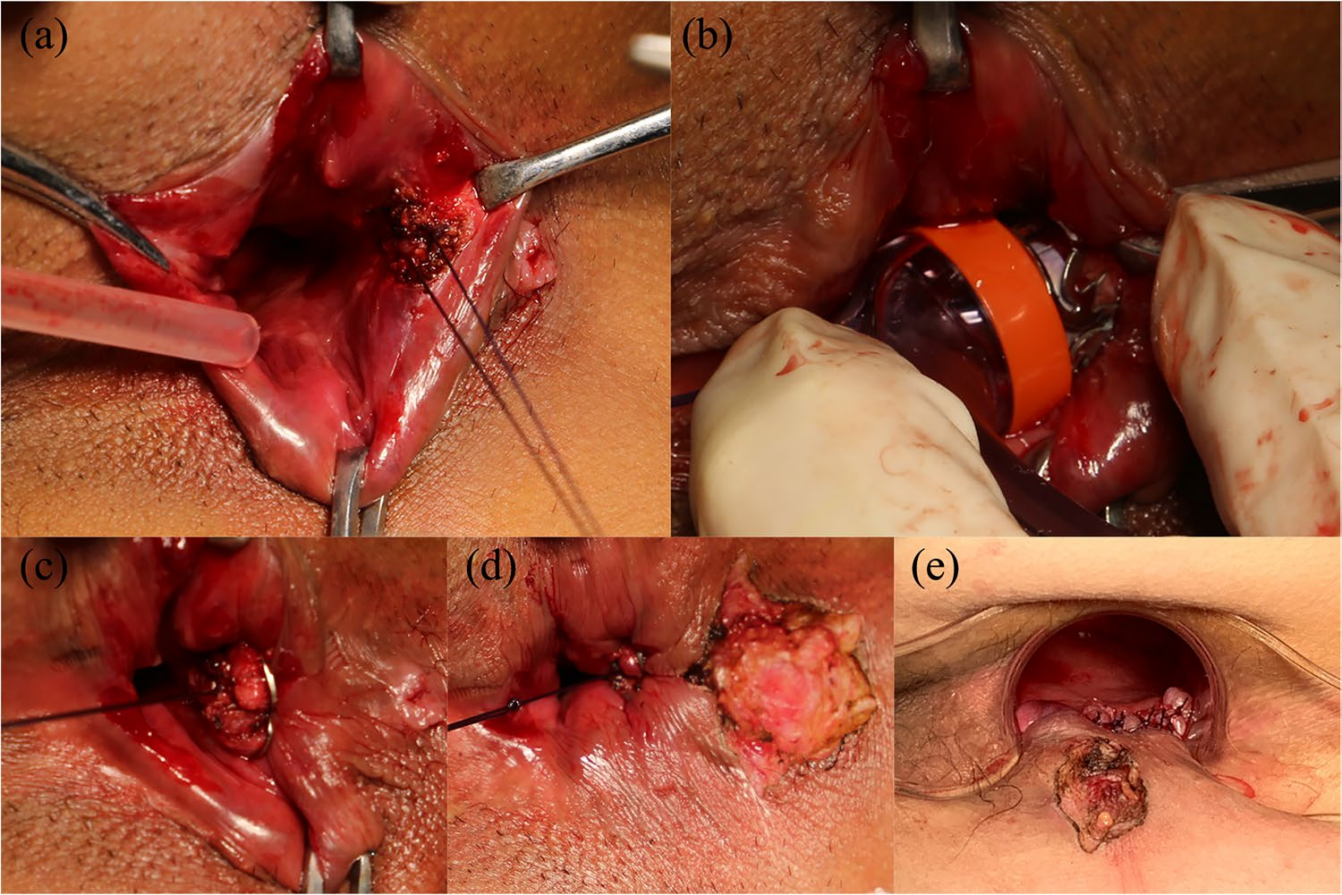
Treatment with AFC. **a** Placement of two U-shaped sutures. **b** Advancement of the clip applicator. **c** Clip release. The clip was applied at the dentate line closing the internal opening. **d** Appropriate removal of the external fistula to ensure adequate drainage. **e** Suture of ERAF

Fifty-one patients with cryptoglandular fistula-in-ano were enrolled between January 2019 and January 2020 in the National Colorectal Disease Center of Nanjing Hospital of Chinese Medicine Affiliated to Nanjing University of Chinese Medicine. The patients were randomly assigned for management with either AFC or ERAF (Fig. 2). The primary endpoint was fistula healing after proctology clip placement assessed by clinical examination (at weeks 6 and 12 and month 6) and endorectal ultrasonography (ERUS) (at week 6). The secondary endpoints included reduced pain score (Visual Analog Scale, VAS) and incontinence (Wexner) score at follow-up. Safety evaluation based on adverse events was also monitored.

## Results

Of the 51 patients, 25 were included in the AFC group and 26 in the ERAF group. There were no significant differences in selected features between the two groups at baseline.

The fistula healing rates within the AFC group and ERAF group at week 6 were 48.0% (12/25) and 46.2% (12/26), respectively, with no significant difference between them (*p* = 1.0). At week 12, the healing rates between the AFC group (88.0%, 22/25) and ERAF group (69.2%, 18/26) did not differ significantly (*p* = 0.1), while at month 6, there was a significant difference (*p* = 0.021) between the AFC (92.0%, 23/25) and the ERAF (65.4%, 17/26) groups. Furthermore, at month 6, the trans-sphincteric fistula healing rate differed significantly (*p* = 0.047) between the AFC (100.0%, 20/20) and the ERAF (80.0%, 15/20) groups (Table 1). There was no significant difference in the VAS and Wexner scores between the two groups. Fewer adverse events were observed in the AFC group (32.0%) than in the ERAF group (53.9%), although the difference was nonsignificant.

**Table 1:** Primary endpoint at month 6

Patients	AFC			ERAF			Analysis		
	Total	healed	%	Total	healed	%	Difference	95%CI	*p* value
All cases	25	23	92.0	26	17	65.4	26.62	3.83–46.63	0.021
Subgroup analysis									
Trans-sphincteric	20	20	100.0	20	15	80.0	20.00	3.78–46.87	0.047
Supra-sphincteric	5	3	60.0	6	2	33.3	26.67	− 25.37–63.50	0.570

## Discussion

Although ERAF does not destroy the anal sphincter and has a moderate success rate, postoperative recurrence and incontinence represent challenges.^1,2^ The AFC exerts constant pressure on the internal opening resulting in permanent and dynamic closure.

In the AFC group, one clip fell off on its own on day 10, while the rest were removed by cutting the lateral hinges of the clip with the special AFC clip cutter at about 3.8 weeks (range, 3–4 weeks) without complaint. Six clips needed to be removed under abnormal circumstances. In the ERAF group, eight patients still complained of persistent secretions around the anus at week 12, and five received a second operation. In this study, six cases in the two groups retained a narrow tract without secretion while the internal opening was closed. The key to success was the capture of sufficient tissue volume. If sufficient external drainage cannot be guaranteed, inflammatory complications are inevitable.

The main limitations of this study were the small sample size and the short follow-up time. Nevertheless, the study provides evidence for the safety and efficacy of AFC in the treatment of cryptoglandular fistula-in-ano. AFC is an innovative contribution to minimally invasive sphincter-preserving technology.

## Data Availability

The datasets used and/or analyzed during the current study are available from the corresponding author upon request. After the completion of the trial, all data will be uploaded to China’s clinical trial registration center.

## References

[1] Soltani A, Kaiser AM (2010). Endorectal advancement flap for cryptoglandular or Crohn's fistula-in-ano. Dis Colon Rectum..

[2] Gottgens KW, Smeets RR, Stassen LP (2015). Systematic review and meta-analysis of surgical interventions for high cryptoglandular perianal fistula. Int J Colorectal Dis..

